# Water sanitation in Karachi and its impact on health

**DOI:** 10.1016/j.amsu.2022.103688

**Published:** 2022-04-28

**Authors:** Prince Kumar, Fatima Arshad, Sean Kaisser Shaheen, Arsalan Nadeem, Zarmina Islam, Mohammad Yasir Essar

**Affiliations:** a Faculty of Medicine, Dow University of Health and Sciences, Pakistan; b Faculty of Medicine, Allama Iqbal Medical College, Lahore, Pakistan; c Kabul University of Medical Sciences, Kabul, Afghanistan

**Keywords:** Water sanitation, Hygiene, Water-borne diseases, COVID-19, Karachi, Pakistan

## Abstract

Karachi is the largest city and premiere industrial and financial center of Pakistan yet is subjected to major infrastructure deficits. Of primary concern is poor water sanitation which has predisposed a weak healthcare system and its citizens to increased infectious diseases. In Karachi, causes of this include a mismanaged sewage system, poor urban planning, and overcrowding. Several reasons such as lack of funding, corruption, and mismanagement have exacerbated circumstances placing extra strain on Karachi's already scarce water supply. In addition, lack of maintenance and regulation of the existing system has led to increased contaminated water delivery to citizens. Consequently, outbreaks of various water borne diseases such as typhoid, *helicobacter pylori*, malaria, diarrhea etc have severely impacted healthcare of citizens, especially with the COVID-19 pandemic. The situation is worsened by Karachi's rapidly growing population and lack of awareness among citizens regarding safe drinking water. Prior socioeconomic disparities, and illiteracy complicate access to medications and appropriate healthcare services. However, despite weak efforts from the government, several national and international organizations continue to strive to improve access to clean, drinkable water.

## 1 Introduction

Water sanitation is the process of cleaning and filtering water for the purposes of making it safe for drinking, bathing, cooking etc [1]. According to the World Health organization (10.13039/100004423WHO) and the 10.13039/100004420United Nations Children's Fund (10.13039/100006641UNICEF), globally, as of 2020, around 1 in 4 people were determined to be lacking access to potable water in their place of residence [2]. Particularly, in Pakistan, an estimated 70% of households drink water contaminated with bacteria [3]. However even in Pakistan, there are differences between rural and urban environments in general and even differences between urban environments themselves. In Karachi for example, data gathered by research officers of the Pakistan Council of Research in Water Resources (PCRWR) estimates that up to 91% of drinking water is unsafe to drink [4]. This is in stark contrast to the data collected by the same council in which they found that only 62% of drinking water out of the 29 major cities tested across Pakistan to be unfit for consumption [5]. Furthermore, there are differences in respect to time and in this case an unfortunate one, as a current primary concern is the increased contamination of water through the years. This is evidenced by PCRWR reports conducted in 2015–2016 which find that only 57% of water samples in major cities were contaminated [5].

Poor water sanitation is a cause of concern for a multitude of reasons. For example, in Pakistan, about 50% of all diseases and 40% of deaths occur due to poor quality of drinking water [6]. Secondly, the leading cause of death in infants and children is diarrhea, a waterborne disease. The root cause of waterborne diseases is water with the addition of municipal sewage and industrial waste at various sections of the water distribution network. This coupled with the inefficient and sometimes nonexistent water disinfection and water quality checks at treatment plants [6] are major contributing factors to the high rates of water contamination. Given the circumstances of the pandemic, prior sanitation issues, and the lack of adequate research, it is imperative to address the health impacts of the deteriorating environmental conditions. This is especially so within the context of Karachi where the growing population needs and governmental dysfunction at the municipal level is expected to worsen the already pre-existing health burdens. Therefore, this article's aim is to bring awareness to the present issues regarding the poor sanitation standard and practices in Karachi, Pakistan, as well as the current efforts surrounding water sanitation and lastly identify challenges with corresponding recommendations to tackle the issues.

## 2 Challenges

Multiple factors, ranging from poor infrastructural management to incompetent local authorities, have led to inadequate water sanitation in Karachi.

Lack of appropriate infrastructure along with the increasing population and corruption amongst officials appear to be the most significant factors contributing to Karachi's water problems. Karachi Water and Sewage Board (KWSB) supplies 665 MGD (Millions of Gallons per Day) of water while the estimated demand is 820–1200 MGD [7]. Moreover, this gap is expected to grow along with the city's growing population, and to meet the increasing demand many illegal water hydrants have been installed across the city. KWSB officials report these illegal hydrants have led to theft of 30 million gallons of water along with revenue losses of around Rs 1.3 billion annually [8]. To make matters worse, some KWSB officials have been criticized for taking bribes and underreporting many of the illegal hydrants which has masked the true economic impacts of such theft [9].

Leakages, ineffective management, and lack of future planning are all serious obstacles faced by water sanitation services in Karachi. Numerous household and drinking water pipelines run parallel to sewage discharge pipelines and occasional leakages are caused by lack of regular cleaning, especially post monsoon season [10]. This has led to the bacterial contamination of an already scarce water supply, indicating poor management and incompetency of local authorities [10].

Microbial and chemical contamination have made the situation worse, and chemical waste from factories along with agricultural run-off such as pesticides and fertilizers further pollute the city's water. Reports indicate that 31 out of 107 samples of ground water had a pesticide count beyond the permissible limits set by World Health Organization (WHO) [11]. In addition to chemical contaminants, high levels of *Escherichia coli* (E.coli) were found in Karachi's water sources and the city's water was labelled as the most contaminated water in all of Sindh by Pakistan Council of Research in Water Resources' (PCRWR) research teams [12]. This excessive contamination of groundwater means it requires treatment before it is safe for consumption and yet only 62% households receive water with any prior treatment while 1.91 of the 2.16 million cubic meters (mcm) of wastewater remains untreated on a daily basis [10]. Furthermore, many residents have complained that the water tastes salty or looks cloudy and have often found insects crawling in it. This has led many to purchase bottled water from private organizations [12].

Socio-economic disparities make affordability of bottled water a challenge, especially for 8.9% of Karachi's population which lives below the poverty line [13]. The situation is worse in overcrowded and unplanned settlements like Orangi, which accommodates most of the city's poor population [12]. The dire situation is indicated in reports which show that some neighborhoods in Orangi did not receive water from the main pipelines for 33 days [12].

The inadequate sanitation along with a growing population has led to many adverse health impacts for Karachi's citizens, such as the increased spread of many diseases like typhoid fever, the causative organism of which is found mainly in contaminated water and undercooked food [14]. There has been a recent surge in extensive drug-resistant typhoid cases and overcrowded slums with poor sanitation infrastructure prove to be ideal breeding grounds for these microorganisms [14]. The situation is further complicated by the spread of COVID-19, which presents with symptoms similar to typhoid fever, making differential diagnosis difficult [15]. As previously stated, Karachi's water is highly contaminated with E. coli which increases the prevalence of diarrhea. This along with high figures of open defecation (25 million) in Pakistan further contaminate the water [16]. Open defecation could further accelerate the spread of diseases like rotavirus and *Helicobacter pylori*, both of which are transmitted by direct or indirect contact with an infected person's feces [17,18]. Lastly, diseases like malaria and dengue can also spread through contaminated water as the vectors for these diseases, *Anopheles* and the *Aedes* mosquitos respectively, breed in pools of stagnant water [19,20]. Overall these water-borne diseases exert a significant burden on the city's healthcare system and urgent efforts must be made to improve the sanitation services in Karachi.

## 3 Efforts and recommendations

In accordance with the SDGs, Pakistan's vision 2025 addresses the plights of water security and aims at increasing water storage capacity, with improved agricultural efficiency and availability of clean drinking water [21]. Goal six of the Sustainable Development Goals (SDGs) calls for ensuring availability and sustainable management of water and sanitation for all, water use efficiency, and integrated water resources management. Nevertheless, inadequate efforts have been made to prevent the consequences of Karachi's water crisis [22].

Of note, a campaign titled “#Fixit” founded by a social worker, Alamgir Khan was started in January 2016 in Karachi and developed into one of the fastest developing NGOs [23]. It highlights and advocates for solutions for social, civil and political issues faced by the community. The campaign has highlighted water and sewerage issues in distinctively creative ways involving the government officials to raise awareness amongst the masses [24]. Another NGO titled “Hisaar Foundation” has been operating since the past 14 years with extensive work done on building the cooperation to shift the water paradigm through a “think tank” on rational use of water, universities for water network, and several programs on low-cost solutions with relevance to the water-food-livelihood nexus [25]. WaterAid is an international NGO that established its first office in Pakistan in 2006 and provides technical assistance to the government for clean water, proper sanitation and good hygiene [26].

According to a citizen report card, residents of most towns of Karachi suggested that the Karachi Water and Sewerage Board (KWSB) should first prioritize water availability and then address water cleanliness [27]. The chronic problems of supply and distribution must be addressed to avoid further pressures on a worsening system. Major reforms in leadership and recruitment policies must be adopted to ensure modern water utility are run by trained personnel [28].

Residents heavily contribute to the problems of distribution and contamination of water supply by “stealing” water with barely any identifiable trace of theft due to punctures in the mainlines which run under the ground [22]. Sewerage gets mixed into the water through these punctured points ultimately contaminating the supply at a large scale. Residents must ensure periodical cleaning of water tanks to avoid the risks of such contamination. Some areas lack supply of water for many days or weeks at stretch [22]. Such damaged pipes must be replaced swiftly by the water board as the insides of these pipes develop biofilms which are perfect breeding grounds for bacteria that multiply exponentially in a short period of time [22].

Water filtration and desalination plants in coastal areas should be installed to compensate for the exponentially increasing water shortage. A good example would be the solar-powered plant established near Mubarak Village, some 42 km from the heart of Karachi, which started producing clean drinking water at a capacity of 5000 L per day [29]. UNICEF proposes water sanitation and hygiene (WASH) to ensure a safe and clean community with adequate water and sanitation for every child [30]. Solutions under WASH include ending open defecation through communities accepting toilet usage as a regular habit in their lives, improving quality of water through providing support to the government to build frameworks, and promoting WASH in schools as well as health facilities to ensure healthy habits such has hand washing among students [30].

## 4 Conclusion

Karachi's water supply and sewage system needs immediate attention from the government and adequate planning to accommodate the rapidly increasing population and urbanization of the city to avoid a severe crisis in the future.

## Ethical approval

N/A.

## Sources of funding

None.

## Authors' contributions

All authors equally contributed.

## Registration of research studies

N/A.

Name of the registry:

Unique Identifying number or registration ID:

Hyperlink to your specific registration (must be publicly accessible and will be checked):

## Guarantor

N/A.

## Consent

N/A.

## Conflicts of interest

None declared.
